# Exercise Dependence and Its Health Correlates: A Cross‐Sectional Survey‐Based Study of Impulsivity, Depression Symptom Severity, and Exercise Duration

**DOI:** 10.1002/hsr2.73162

**Published:** 2026-09-07

**Authors:** Beyza Nur Gürbüz, Hasan Durmuş, Yasin Kavla, Mark D. Griffiths, Attila Szabo

**Affiliations:** ^1^ Department of Public Health Erciyes University Kayseri Türkiye; ^2^ Department of Psychiatry, Cerrahpasa Medical Faculty Istanbul University‐Cerrahpasa Istanbul Türkiye; ^3^ Nottingham Institute of Psychology Nottingham Trent University Nottingham UK; ^4^ Faculty of Health and Sport Sciences Széchenyi István University Győr Hungary

**Keywords:** depression, exercise dependence, exercise duration, impulsivity, mental health

## Abstract

**Background and Aims:**

Exercise dependence is a potentially maladaptive pattern in which regular training becomes compulsive, poorly controlled, and associated with functional impairment. Given uncertainty about whether dependence‐related symptoms are more closely associated with affective distress, impulsivity, or exercise exposure, the present study examined multidimensional impulsivity, depressive symptom severity, sociodemographic characteristics, and weekly exercise duration in relation to the 21‐item Exercise Dependence Scale (EDS‐21) risk classification among regular exercisers.

**Methods:**

In the present cross‐sectional study, 850 adults aged ≥18 years who regularly exercised at municipal centers, sports facilities, and healthy‐living complexes in Türkiye completed the EDS‐21, Short UPPS‐P Impulsive Behavior Scale, Short Beck Depression Inventory, and questions on weekly exercise duration, sociodemographic information, and health‐related variables. Analyses included descriptive statistics, Spearman correlations, Kruskal‐Wallis tests, Firth‐penalized binary logistic regression, and an exploratory multinomial logistic regression retaining all three EDS‐21 categories.

**Results:**

Thirty‐two participants met the EDS‐21 dependence‐risk classification (3.8%), while 443 were symptomatic (52.1%) and 375 were asymptomatic (44.1%). EDS‐21 total score correlated with sensation seeking (*r* = 0.28, *p* < 0.001), total impulsivity (*r* = 0.23, *p* < 0.001), and depression symptom severity (*r* = 0.09, *p* = 0.01). In the Firth model, being single (OR = 5.77, 95% CI [1.23, 33.03], *p* = 0.02), higher total impulsivity (OR = 1.71 per 10‐point increase, 95% CI [1.07, 2.75], *p* = 0.02), and longer weekly exercise duration (OR = 1.23 per additional 60 min/week, 95% CI [1.14, 1.33], *p* < 0.001) were associated with the dependence‐risk classification. Depression symptom severity was not (*p* = 0.25).

**Conclusion:**

EDS‐21 dependence‐risk classification was associated with impulsivity, single marital status, and weekly exercise duration in the present sample. The null depression finding should be interpreted cautiously because scores were low and showed limited between‐group variability. These cross‐sectional findings identify correlates rather than temporal or causal predictors.

## 1 Introduction

Regular and sustained participation in physical exercise is widely recognized for its physiological and psychological health benefits, serving as a cornerstone for well‐being and the prevention of various chronic conditions [1, 2, 3, 4, 5]. Early work by Glasser [6] identified intensive physical activity as a “positive addiction” that can bolster individual strength and foster healthy lifestyles [7]. However, this adaptive behavior could become maladaptive, exemplifying the “exercise paradox” in which physical activity transitions from a therapeutic habit to a compulsive, potentially dysfunctional pattern [7, 8].

Exercise dependence is operationalized in the present study as a multidimensional maladaptive behavioral problem that could lead to behavioral addiction (exercise addiction) characterized by loss of control, compulsive engagement, and physiological or psychological withdrawal symptoms with eventual clinically significant impairment in health, social relationships, and professional life [1, 3, 8, 9]. However, dependence is only one pillar in exercise addiction, the other being compulsion [10]. Therefore, the term “addiction” is not used in the context of the present study. Despite exercise being a socially accepted and widely encouraged behavior, the individual's inability to regulate exercise volume, often persisting despite severe musculoskeletal injuries or social deterioration, demonstrates its potentially dysfunctional nature [11, 12].

Contemporary theory‐driven frameworks, most notably Egorov and Szabo's [7] interactional model, posit that exercise addiction, including its exercise dependence component, emerges from an idiographic “black box” interaction between specific psychological vulnerabilities and stressful life events [3, 7]. These vulnerabilities are increasingly associated with specific personality traits, such as perfectionism and neuroticism, as well as multidimensional impulsivity, particularly negative urgency [1, 2, 12].

Exercise dependence also exhibits high comorbidity with other risk behaviors, particularly eating disorders, where the risk of co‐occurrence is estimated to be more than three and a half times greater than in the general population [3]. Despite nearly half a century of empirical scrutiny and established associations with motivational regulations and affective hedonism, exercise dependence, as a pillar of exercise addiction, remains excluded from diagnostic manuals such as the latest (fifth edition) of the *Diagnostic and Statistical Manual of Mental Disorders* (DSM‐5) due to ongoing methodological heterogeneity and a lack of standardized diagnostic criteria [8, 9, 13].

For a thorough understanding, in the present paper, the term “exercise dependence” is used rather than “addiction” because the instrument used (i.e., the 21‐item Exercise Dependence Scale [EDS‐21]) assesses dependence‐like symptom clusters, including tolerance, withdrawal, loss of control, and continuance despite adverse consequences. Addiction is a broader diagnostic and theoretical construct that typically implies persistent compulsive/reward‐driven behavior with clinically significant impairment [10], whereas dependence in the present study denotes elevated self‐reported dependence symptoms rather than a confirmed psychiatric diagnosis. This distinction is important for health science practice because it helps avoid pathologizing high‐volume training while still identifying exercisers who may benefit from assessment, education, or referral. Accordingly, the EDS‐21 at‐risk category is referred to throughout the present paper as the “dependence‐risk classification.” This denotes a psychometric classification and not a confirmed clinical diagnosis.

One of the most significant challenges in exercise dependence research is the lack of a formal clinical diagnosis and standardized classification in major psychiatric reference manuals such as the DSM‐5 and the eleventh revision of the *International Classification of Diseases* (ICD‐11) [7, 14]. This absence has resulted in a wide range of reported prevalence rates, with estimates for individuals “at‐risk” ranging from 0.3% in the general population to 77% among specific endurance cohorts [13, 15]. Scholars attribute these inconsistencies to the heterogeneous use of psychometric screening instruments, most notably the Exercise Addiction Inventory (EAI [16]) and the Exercise Dependence Scale (EDS [17]), which produce scalar risk scores rather than definitive clinical diagnoses [18, 19]. These instruments may overpathologize dedication, because elite athletes may interpret scale items as expressions of commitment and passion rather than behavioral morbidity [8, 14, 19].

Moreover, the distinction between primary exercise dependence and secondary or instrumental exercise dependence—recently termed “instrumental exercise”—remains a major methodological hurdle [3, 7]. In secondary cases, excessive training functions as an instrumental means to achieve non‐exercise goals, typically related to eating disorders or body image disturbances, where the risk for addictive patterns is substantially elevated [20, 21, 22, 23]. These nuances emphasize that current prevalence figures likely capture a broad spectrum of dedication and psychopathology, necessitating validation through theory‐driven research and in‐depth clinical interviews [14, 18].

Building upon the distinction between primary and secondary exercise dependence, it is important to recognize the extensive psychiatric comorbidities associated with this potentially dysfunctional behavior. Recent meta‐analytic evidence confirms that exercise dependence is significantly associated with a spectrum of mental health problems, including anxiety, obsessive‐compulsive tendencies, stress, and, notably, depression [22]. In fact, clinical assessments have shown that major depressive disorder is highly prevalent among individuals at‐risk for exercise dependence, with excessive exercise patterns often preceding the onset of depressive episodes rather than merely resulting from them [24].

Exercise dependence has also been associated with specific personality traits and multidimensional psychological factors. Predictive modeling studies have identified perfectionism, alongside drives for muscularity and thinness, as important correlates of higher exercise‐dependence scores and risk classifications [25]. These findings support examining individual differences such as impulsivity and depression symptom severity when distinguishing highly committed exercise from potentially dysfunctional dependence‐related patterns.

Considering these conceptual hurdles, recent theory‐driven research has increasingly focused on psychological and sociodemographic correlates that may help distinguish elevated dependence‐related symptoms from high commitment. Multidimensional impulsivity has emerged as a relevant correlate in this context. More specifically, facets such as negative urgency (the tendency to act rashly under emotional distress) and sensation seeking have been associated with higher exercise‐dependence risk [1]. Concurrently, exercise dependence has been associated with severe mood disturbances, particularly major depressive disorder, although the strength and temporal direction of this relationship remain uncertain across study designs [22, 24].

Recent predictive modeling also indicates that psychological characteristics co‐occur with behavioral metrics and sociodemographic factors in multivariable exercise‐dependence risk profiles [25]. Digital and sociocultural factors may also be relevant. Among emerging adults, problematic social media use, fitspiration exposure, and narcissistic traits have been associated cross‐sectionally with higher exercise addiction risk [26]. Because these data were cross‐sectional, the direction and temporal ordering of these associations remain unclear. Despite this growing evidence, studies simultaneously examining the independent associations of multidimensional impulsivity, depression symptom severity, sociodemographic characteristics, and weekly exercise duration with an EDS‐21 dependence‐risk classification remain scarce.

Therefore, to address these gaps, the present cross‐sectional study examined whether, among adult regular exercisers, multidimensional impulsivity, depression symptom severity, sociodemographic characteristics, and weekly exercise duration were independently associated with EDS‐21 dependence‐risk classification. Based on reward‐sensitivity, affect‐regulation, and behavioral‐exposure frameworks, it was hypothesized that higher impulsivity and depression symptom severity would be positively associated with exercise dependence (H_1_), and that longer weekly exercise duration would remain independently associated with the dependence‐risk classification after adjustment for sociodemographic and health‐related variables (H_2_).

## 2 Method

### 2.1 Research Design, Participants, and Procedure

A cross‐sectional survey design was employed to examine factors associated with exercise dependence among regular exercisers. The study population comprised individuals aged 18 years and older who actively exercised at various municipal social, sports, and healthy‐living complexes in a major urban center in Türkiye (Table 1). Their exercise routine included running, walking, cycling, swimming, fitness, Pilates, yoga, martial arts, kayaking, rowing, and volleyball. To maximize representativeness and ensure a diverse sample, researchers visited multiple facilities at different times of the day to collect data through face‐to‐face surveys. Ethical approval was obtained from the Erciyes University Ethics Committee (Approval No: 2025/196; April 16, 2025). Signed written informed consent was obtained at the time of survey administration. The analytical dataset contained no direct personal identifiers. Reporting was aligned with the STROBE recommendations for cross‐sectional observational studies for transparency and reproducibility in health sciences research [27].

**Table 1:** Sociodemographic, behavioral characteristics, and baseline scale scores of the participants (*n* = 850).

Variables	n/(mean)	%/(SD)	Variables	n/(mean)	%/(SD)
Sociodemographic characteristics					
Gender			Behavioral characteristics		
Male	434	51.1	Alcohol use (Yes)	128	15.1
Female	416	48.9	Cigarette smoking (Yes)	186	21.9
Age group			Internet gaming (Yes)	311	36.6
18‐44 years	689	81.1	EDS‐21 exercise dependence classification		
45 years and older	161	18.9	Asymptomatic	375	44.1
Education level			Symptomatic	443	52.1
High school and below	222	26.2	Dependence‐risk	32	3.8
University and above	628	73.8	Continuous variables		
Employment status			Years of exercise	3.60	(4.75)
Student	272	32.0	Weekly exercise duration (minutes)	254.35	(182.21)
Employed	381	44.8	EDS‐21 Total Score	47.41	(17.67)
Unemployed/retired	197	23.2	UPPS Total Score	41.77	(8.27)
Marital status			Depression (SBDI) Score	3.16	(3.03)
Single	494	58.1			
Married	356	41.9			

*Note:* For continuous variables, values are presented as mean and standard deviation (SD); for categorical variables, values are presented as *n* (%).

Abbreviations: EDS‐21, Exercise Dependence Scale‐21; UPPS, UPPS‐P Impulsive Behavior Scale; SBDI, Short Beck Depression Inventory; SD, standard deviation.

### 2.2 Sample Size Calculation

The sample size was determined *a priori* using G*Power software (version 3.1.9.2). Assuming a low effect size, an alpha level of 0.05, and a statistical power of 0.80 for a two‐tailed Pearson correlation analysis between exercise dependence and impulsive behavior, the minimum required sample size was calculated to be 782 [28]. Accounting for a potential 10% data loss (e.g., missing or incomplete responses), the target sample size was 850 participants. This calculation pertained to the primary correlation‐based objective and should not be interpreted as establishing adequate information for a rare‐outcome multivariable logistic model. Because only 32 participants met the EDS‐21 dependence‐risk classification, the effective information for the binary multivariable analysis was limited. This was addressed using bias‐reduced Firth‐penalized logistic regression and conservative interpretation [29, 30].

### 2.3 Measures

Data were collected using a multi‐section survey instrument comprising a sociodemographic information form and three standardized psychometric scales.

*Sociodemographic and Behavioral Information Form*: These questions were created by the research team to gather data on participants' age (in years), gender (male or female), and exercise habits. Participants reported their exercise preferences using a multiple‐response item that allowed selection of multiple activities, such as swimming, running, and martial arts. They also provided information on current alcohol consumption (yes or no) and smoking status (yes, no, or quit).

*Exercise Dependence Scale‐21* (EDS‐21): Exercise dependence was assessed using the 21‐item EDS‐21 [17], adapted into Turkish by Gürbüz and Aşçı [31]. The EDS‐21 assesses exercise dependence symptoms over the previous 3 months, with items (e.g., “I exercise to avoid feeling irritable”) rated on a six‐point Likert scale ranging from 1 (*never*) to 6 (*always*). Higher scores indicate greater exercise dependence symptoms. Participants are classified as (i) at‐risk for exercise dependence if they score in the dependent range (typically 5–6), on at least three of the seven dependence criteria, (ii) non‐dependent symptomatic if they score in the symptomatic range (typically 3–4), on at least three criteria, or show a combination of dependent‐ and symptomatic‐range scores without meeting the at‐risk threshold, or (iii) non‐dependent asymptomatic if they score in the asymptomatic range (typically 1–2), and do not meet criteria for the other two categories. The Turkish version supports a five‐factor structure comprising time and exercise preference, lack of control, withdrawal effects, tolerance, and continuance. In the present study, Cronbach's alpha (α) for the EDS‐21 was 0.93. In the present report, the EDS‐21 at‐risk category is termed the “dependence‐risk classification” to emphasize that it is a psychometric classification rather than a confirmed clinical diagnosis.

*Short Beck Depression Inventory (SBDI*): Depression symptom severity was assessed using the seven‐item Short Beck Depression Inventory, also known as the Beck Depression Inventory for Primary Care (BDI‐PC [32]), adapted into Turkish by Özdemir and Dağdeviren [33]. The scale comprises seven cognitive and affective depression symptoms, and each item (e.g., “I feel sad”) is rated on a four‐point Likert scale ranging from 0 (*absence of the symptom*) to 3 (*severe/full‐blown presence of the symptom*). Total scores range from 0 to 21, with higher scores indicating greater depression symptom severity. A score ≥4 is used as the recommended screening cut‐off, and severity categories are defined as minimal (0–3), mild (4–6), moderate (7–9), and severe (10–21). In the present study, internal consistency of the SBDI was acceptable (Cronbach's α = 0.77).

*Short UPPS‐P Impulsive Behavior Scale:* Multidimensional impulsivity was assessed using the 20‐item short version of the UPPS‐P Impulsive Behavior Scale [34]; Turkish version: Fournier et al. [35]. The scale assesses five facets of impulsivity, each represented by four items: negative urgency, positive urgency, lack of premeditation, lack of perseverance, and sensation seeking. Items (e.g., “I generally like to see things through to the end”) are rated on a four‐point Likert scale from 1 (*agree strongly*) to 4 (*disagree strongly*), and relevant items are reverse‐coded so that higher scores indicate greater impulsivity. The scale has no clinical cut‐off score. In the present study, internal consistency for the total score was acceptable (Cronbach's α = 0.77).

### 2.4 Data Analysis

Data were initially analyzed using *IBM SPSS Statistics, Version 28.0*. Before the main analyses, the data were examined for missing values, outliers, and violations of the normality assumption. Because the continuous variables (EDS‐21, UPPS‐P, and SBDI scores) did not meet the assumption of normality (assessed using the Kolmogorov‐Smirnov/Shapiro‐Wilk tests and skewness/kurtosis values), nonparametric methods were used. Descriptive statistics for sociodemographic, behavioral, and clinical characteristics are presented as frequencies (*n*), percentages (%), means, and standard deviations (SDs). Mann‐Whitney U and Pearson's chi‐square tests were used for supplementary gender comparisons.

To examine bivariate relationships among exercise dependence, impulsivity subscales, and depression symptom severity, Spearman's rank‐order correlation coefficients (rho) were calculated. To compare continuous variables across the three EDS‐21 classification groups (asymptomatic, symptomatic, and dependence‐risk), the Kruskal‐Wallis H test was used. The bivariate analyses and binary multivariable model addressed the prespecified study questions. For the binary multivariable analysis, the dependence‐risk classification was coded 1, and the symptomatic/asymptomatic classifications were combined as 0, consistent with the original primary analysis. Ten prespecified covariates were entered simultaneously: gender, age, marital status, body mass index (BMI), chronic disease status, internet gaming, regular alcohol use, weekly exercise duration, depression symptom severity, and total impulsivity.

Multicollinearity was assessed using variance inflation factors (VIFs), which ranged from 1.04 to 2.34. Because the binary outcome included only 32 dependence‐risk cases (approximately 3.2 events per candidate covariate), conventional maximum‐likelihood estimates with stepwise selection were considered vulnerable to sparse‐data bias, coefficient instability, and overfitting. Therefore, the revised primary binary model used Firth‐penalized logistic regression with all 10 covariates entered simultaneously [29]. Adjusted odds ratios (ORs) are reported with 95% profile penalized‐likelihood confidence intervals (CIs) and two‐sided profile‐likelihood ratio *p*‐values. As a complementary exploratory analysis, multinomial logistic regression retained all three EDS‐21 categories, with the asymptomatic group as the reference, to examine whether associations differed for the symptomatic and dependence‐risk classifications. The dependence‐risk contrast remained exploratory because that category contained only 32 participants. Revised regression analyses were performed in *Python 3.13.5* using *NumPy 2.3.5, SciPy 1.17.0*, and *statsmodels 0.14.6*. All statistical tests were two‐sided, with an *a priori* alpha level of 0.05.

## 3 Results

The sociodemographic, behavioral, and baseline clinical characteristics of the participants (*n* = 850) are summarized in Table 1. The sample was relatively balanced in terms of gender (51.1% male) and consisted predominantly of young adults aged 18–44 years (81.1%). Based on the EDS‐21 classification, the two larger proportions of participants were categorized as either symptomatic (52.1%) or asymptomatic (44.1%), while a small number (*n* = 32; 3.8%) met the EDS‐21 dependence‐risk classification. Additionally, specific comparisons of scale scores by gender, along with the distribution of primary exercise types engaged in by the participants, are detailed in Supplementary Information S1: Tables 1 and 2.

Bivariate relationships among the study variables were examined using Spearman's rank‐order correlations (Table 2). Exercise dependence was significantly and positively associated with both total impulsivity (*r* = 0.23, *p* < 0.001) and depression symptom severity (*r* = 0.09, *p* = 0.01). Among the impulsivity subscales, sensation seeking (*r* = 0.28, *p* < 0.001) and positive urgency (*r* = 0.15, *p* < 0.001) exhibited the strongest correlations with exercise dependence, whereas lack of perseverance showed no significant relationship.

**Table 2:** Correlations between exercise dependence (and subscales), impulsivity, and depression symptom severity.

Variables	(1)	(a)	(b)	(c)	(d)	(e)	(2)
(1) EDS‐21 (Total score)							
a. 2. Time and exercise preference	0.87**	—					
b. 3. Lack of control	0.73**	0.60**	—				
c. 4. Withdrawal effects	0.66**	0.43**	0.39**	—			
d. 5. Tolerance	0.75**	0.53**	0.44**	0.45**	—		
e. 6. Continuance	0.62**	0.50**	0.43**	0.32**	0.38**	—	
(2) UPPS Impulsivity (Total score)	0.23**	0.19**	0.24**	0.19**	0.13**	0.20**	—
(3) Depression symptom severity	0.09*	0.03	0.10*	0.19**	0.00	0.05	0.26**

*Note:* Letters denote the subscales of the Turkish EDS‐21 (1); *p < 0.05, **p < 0.001. Correlation coefficients represent Spearman's rho (*r*) values. Time/exercise preference refers to a single EDS‐21 subscale that assesses time devoted to exercise and preference for exercise over other activities.

To further evaluate these relationships, impulsivity and depression symptom severity levels were compared across the three exercise dependence groups (Table 3). The results showed a statistically significant difference in total impulsivity (*p* < 0.001), with the dependence‐risk group consistently reporting the highest median scores. Medians and interquartile ranges are reported because the group comparisons were conducted using the nonparametric Kruskal‐Wallis H test, which is appropriate when continuous variables are not normally distributed. Significant differences were also observed in positive urgency and sensation seeking, as well as in all EDS‐21 subscales (*p* < 0.001). Conversely, depression symptom severity levels (*p* = 0.29) and the remaining impulsivity subscales did not show significant variations across the exercise dependence categories.

**Table 3:** Comparison of UPPS, EDS‐21 Subscales, and Depression Symptom Severity Levels According to Exercise Dependence Groups.

Variables	Asymptomatic (*n* = 375) Median (Q1–Q3)	Symptomatic (*n* = 443) Median (Q1–Q3)	Dependence‐risk (*n* = 32) Median (Q1–Q3)	*p*
Depression symptom severity	2.0 (1.0–4.0)	3.0 (1.0–5.0)	3.0 (0.0–6.2)	0.29
UPPS impulsivity (total score)	40.0 (34.0–46.0)	43.0 (37.0–48.0)	47.0 (40.5–54.0)	< 0.001
Lack of premeditation	6.0 (4.0–8.0)	6.0 (4.0–8.0)	6.0 (4.0–10.0)	0.34
Positive urgency	10.0 (8.0–12.0)	10.0 (9.0–12.0)	11.0 (9.0–14.0)	0.001
Sensation seeking	10.0 (7.0–12.0)	11.0 (9.0–13.0)	12.0 (10.0–14.0)	< 0.001
Negative urgency	8.0 (6.0–10.0)	8.0 (6.0–10.0)	9.0 (5.8–12.2)	0.05
Lack of perseverance	6.0 (4.5–8.0)	6.0 (5.0–8.0)	7.0 (4.8–8.2)	0.62
EDS‐21 subscales				
Time and exercise preference	14.0 (11.0–17.0)	23.0 (20.0–27.0)	40.5 (35.0–45.2)	< 0.001
Lack of control	3.0 (3.0–4.0)	7.0 (4.0–9.0)	15.0 (9.8–18.0)	< 0.001
Withdrawal effects	5.0 (3.0–7.0)	9.0 (7.0–12.0)	17.5 (15.0–18.0)	< 0.001
Tolerance	5.0 (3.0–8.0)	9.0 (8.0–12.0)	16.5 (15.0–18.0)	< 0.001
Continuance	3.0 (3.0–4.0)	5.0 (3.0–8.0)	10.5 (3.0–16.0)	< 0.001

*Note:* Depression symptom severity was low overall (mean = 3.16, SD = 3.03), and median SBDI scores were similar across the asymptomatic, symptomatic, and dependence‐risk groups (2.0, 3.0, and 3.0, respectively), indicating limited between‐group variability and a possible floor effect.

Given the small number of dependence‐risk cases (*n* = 32), the prespecified 10‐covariate binary model was re‐estimated using Firth‐penalized logistic regression rather than backward stepwise maximum‐likelihood selection (Table 4). Single marital status, higher total impulsivity, and longer weekly exercise duration were associated with the EDS‐21 dependence‐risk classification in the penalized model, whereas the other covariates were not independently associated.

**Table 4:** Firth‐penalized logistic regression for the EDS‐21 dependence‐risk classification.

Predictors	Unit/contrast	Adjusted OR	95% profile CI	*p*
Gender	Male vs. female	1.16	[0.51, 2.73]	0.73
Age	Per 1 year	1.02	[0.96, 1.08]	0.50
Marital status	Single vs. married	5.77	[1.23, 33.03]	0.02
Body mass index	Per 1 kg/m^2^	0.95	[0.84, 1.05]	0.33
Chronic disease	Yes vs. no	1.22	[0.26, 4.23]	0.76
Internet gaming	Yes vs. no	1.90	[0.85, 4.42]	0.12
Regular alcohol use	Yes vs. no	0.51	[0.13, 1.48]	0.23
Weekly exercise duration	Per +60 min/week	1.23	[1.14, 1.33]	< 0.001
Depression symptom severity	Per +1 SBDI point	1.06	[0.96, 1.17]	0.25
UPPS‐P total impulsivity	Per +10 points	1.71	[1.07, 2.75]	0.02

*Note:* Outcome coding: 1 = EDS‐21 dependence‐risk classification; 0 = asymptomatic/symptomatic combined. All 10 prespecified covariates were entered simultaneously using Firth‐penalized logistic regression; no stepwise variable selection was used. Two‐sided *p*‐values are from profile‐likelihood ratio tests. Because only 32 participants met the dependence‐risk classification, estimates—particularly marital status—remain imprecise and should be interpreted cautiously.

Abbreviations: OR, adjusted odds ratio; CI, profile penalized‐likelihood confidence interval; SBDI, Short Beck Depression Inventory.

**Figure 1:**
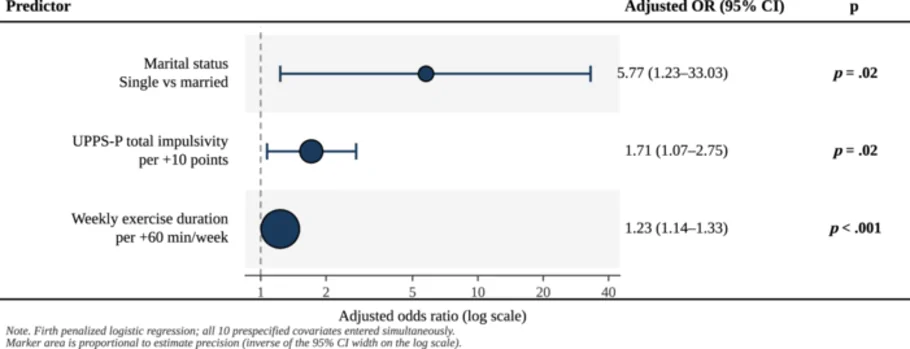
Selected adjusted associations with the EDS‐21 dependence‐risk classification from the Firth‐penalized logistic regression. Odds ratios (ORs) and 95% profile penalized‐likelihood confidence intervals (CIs) are shown. UPPS‐P total impulsivity is shown per 10‐point increase and weekly exercise duration per additional 60 min per week. The markedly wider CI for marital status indicates substantially lower precision than for the continuous covariates; comparisons should therefore consider both effect size and CI width. The vertical line at OR = 1 indicates no association. Full‐model estimates are presented in Table 4.

Participants who were single had higher adjusted odds of the dependence‐risk classification than married participants (OR = 5.77, 95% profile CI [1.23, 33.03], *p* = 0.02), but the wide confidence interval indicated substantial imprecision, and this estimate should be regarded as exploratory. A 10‐point higher UPPS‐P total score was associated with higher odds of being classified in the EDS‐21 dependence‐risk group, relative to the combined asymptomatic and symptomatic groups (OR = 1.71, 95% profile‐likelihood CI [1.07, 2.75], *p* = 0.02). Each additional 60 min of weekly exercise duration was also associated with higher odds of EDS‐21 dependence‐risk classification (OR = 1.23, 95% profile‐likelihood CI [1.14, 1.33], *p* < 0.001). Depression symptom severity was not independently associated with the dependence‐risk classification (OR = 1.06 per point, 95% profile CI [0.96, 1.17], *p* = 0.25). In the complementary multinomial model retaining all three EDS‐21 categories, total impulsivity and weekly exercise duration were associated with *both* the symptomatic and dependence‐risk classifications relative to the asymptomatic group, whereas the single‐marital‐status estimate was elevated only for the dependence‐risk contrast and remained imprecise (Table 5). Selected focal estimates from the Firth model are shown in Figure 1.

**Table 5:** Exploratory multinomial logistic regression retaining all three EDS‐21 classification groups.

Predictors	Unit/contrast	Adjusted OR	95% CI	*p*
Symptomatic vs. asymptomatic				
Gender	Male vs. female	1.40	[1.03, 1.91]	0.03
Age	Per 1 year	0.98	[0.96, 0.99]	0.006
Marital status	Single vs. married	1.14	[0.76, 1.70]	0.53
Body mass index	Per 1 kg/m^2^	0.99	[0.95, 1.03]	0.72
Chronic disease	Yes vs. no	0.62	[0.38, 1.01]	0.05
Internet gaming	Yes vs. no	1.10	[0.79, 1.53]	0.59
Regular alcohol use	Yes vs. no	1.73	[1.13, 2.67]	0.01
Weekly exercise duration	Per +60 min/week	1.15	[1.08, 1.22]	<0.001
Depression symptom severity	Per +1 SBDI point	1.01	[0.95, 1.06]	0.83
UPPS‐P total impulsivity	Per +10 points	1.28	[1.05, 1.56]	0.01
Dependence‐risk vs. asymptomatic				
Gender	Male vs. female	1.46	[0.60, 3.56]	0.40
Age	Per 1 year	1.00	[0.94, 1.07]	0.88
Marital status	Single vs. married	7.38	[1.30, 42.01]	0.02
Body mass index	Per 1 kg/m^2^	0.94	[0.83, 1.05]	0.27
Chronic disease	Yes vs. no	0.76	[0.16, 3.63]	0.73
Internet gaming	Yes vs. no	2.13	[0.89, 5.11]	0.09
Regular alcohol use	Yes vs. no	0.69	[0.19, 2.53]	0.57
Weekly exercise duration	Per +60 min/week	1.38	[1.25, 1.52]	<0.001
Depression symptom severity	Per +1 SBDI point	1.07	[0.95, 1.19]	0.27
UPPS‐P total impulsivity	Per +10 points	2.06	[1.24, 3.41]	0.005

*Note:* Asymptomatic was the reference outcome category. All 10 covariates were entered simultaneously. This complementary analysis was exploratory; estimates for the dependence‐risk contrast should be interpreted cautiously because only 32 participants were in that category.

Abbreviations: CI, Wald confidence interval; OR, adjusted odds ratio; SBDI, Short Beck Depression Inventory.

## 4 Discussion

The present study examined the associations of multidimensional impulsivity, depression symptom severity, sociodemographic characteristics, and weekly exercise duration with EDS‐21 dependence‐risk classification among a relatively large sample of regular exercisers. The findings partially supported H_1_: exercise dependence was positively associated with both total impulsivity and depression symptom severity in bivariate analyses, but only total impulsivity was independently associated with the dependence‐risk classification in the Firth‐penalized logistic regression. More specifically, sensation seeking and positive urgency showed the strongest bivariate impulsivity‐related associations, whereas depression symptom severity did not significantly differ across EDS‐21 classification groups and was not independently associated in the penalized model. H_2_ was supported because longer weekly exercise duration remained independently associated with the dependence‐risk classification after adjustment for sociodemographic and health‐related variables. Overall, the dependence‐risk classification in the present sample was associated with total impulsivity, single marital status, and greater weekly exercise duration, whereas depression symptom severity showed no independent adjusted association. Overall, these cross‐sectional findings describe concurrent associations rather than a transition process. They do not establish that impulsivity, marital status, or exercise duration precedes, drives, or causes the EDS‐21 dependence‐risk classification.

Impulsivity emerged as the most consistent psychological correlate of EDS‐21 dependence‐risk classification in the present study. In line with H_1_, total impulsivity was independently associated with the dependence‐risk classification, while sensation seeking and positive urgency showed the strongest bivariate associations among the impulsivity facets. This pattern is consistent with recent evidence suggesting that problematic exercise is embedded in broader profiles characterized by impulsivity, reward sensitivity, and dysregulated self‐control [1, 25, 36]. More specifically, sensation seeking may reflect a tendency to pursue arousal and novelty, whereas positive urgency reflects rash action during heightened positive emotional states [37, 38]. However, because these UPPS‐P facets were not entered separately into the multivariable model, they should be interpreted as comparatively stronger bivariate correlates rather than robust independent predictors. Moreover, the present design cannot establish a temporal shift from committed to compulsive exercise.

This interpretation is also consistent with recent neurobiological work. A systematic review of neuroimaging studies concluded that exercise dependence is associated with structural and functional alterations in brain regions implicated in reward processing, emotional regulation, and executive control, including the orbitofrontal cortex, anterior cingulate cortex, inferior frontal gyrus, and amygdala [39]. This literature provides a plausible context for the observed association between impulsivity and dependence‐related symptoms, but the present study did not assess neurobiological mechanisms or temporal change.

A particularly noteworthy finding concerns depression symptom severity. Although bivariate analyses showed a small positive association between depression symptom severity and exercise dependence (*r* = 0.09, *p* = 0.01), depression symptom severity did not significantly differ across EDS‐21 classification groups and was not independently associated with the dependence‐risk classification in the Firth model. Importantly, SBDI scores were low overall (mean = 3.16, SD = 3.03), with median scores of 2.0, 3.0, and 3.0 in the asymptomatic, symptomatic, and dependence‐risk groups, respectively. This restricted variability raises the possibility of a floor effect. Therefore, the null adjusted association should not be interpreted as evidence that depression is unrelated to problematic exercise among populations with greater depressive symptom burden.

This interpretation is broadly consistent with recent evidence showing that exercise dependence is positively associated with depression and other mental health problems, while also highlighting substantial conceptual and methodological heterogeneity across studies [14, 18, 22]. In particular, the absence of a formal diagnostic framework and the continued reliance on self‐report screening tools may inflate or blur associations between problematic exercise and affective symptoms, especially when high commitment is difficult to distinguish from true psychopathology [14, 18].

Excessive exercise may function as a maladaptive affect‐regulation strategy among some individuals, which could contribute to associations with depression reported in other samples [22, 24, 40]. However, the present cross‐sectional data cannot establish the direction of this relationship. Moreover, because eating disorder symptoms, body image disturbance, and exercise motives were not assessed, the present study cannot distinguish primary exercise dependence from secondary or instrumental exercise. Consequently, the present study's findings do not establish a predominantly reward‐ and control‐related profile over a secondary form of problematic exercise. This requires studies that directly assess eating pathology, body image, and exercise motives [3, 14].

Regarding sociodemographic factors, single marital status was associated with higher odds of the EDS‐21 dependence‐risk classification in the Firth model (OR = 5.77, 95% profile CI [1.23, 33.03], *p* = 0.02). The confidence interval was very wide, indicating substantial uncertainty around the magnitude of the association. This finding should therefore be regarded as exploratory and hypothesis‐generating rather than as evidence that marital status is the strongest sociodemographic determinant. Recent research suggests that sociodemographic characteristics, including marital status, may be associated with exercise‐dependence risk profiles [41], but larger samples with more dependence‐risk cases are needed.

Possible explanations involving greater discretionary time among single individuals, fitness‐center social environments, social comparison, body‐related reinforcement, or exercise‐related identity were not assessed in the present study. Therefore, these mechanisms should be treated only as hypotheses for future research rather than explanations supported by the present data [23, 42, 43].

Finally, longer weekly exercise duration was associated with higher odds of EDS‐21 dependence‐risk classification in the Firth model (OR = 1.23 per additional 60 min/week, 95% profile CI [1.14, 1.33], *p* < 0.001). This association is consistent with, but does not demonstrate, tolerance‐like processes described in theoretical accounts of exercise dependence [14, 40]. Given the cross‐sectional design, longer exercise duration should be interpreted as a concurrent behavioral correlate rather than evidence of progression toward dependence.

However, high exercise volume alone is not synonymous with pathology. It is more clinically informative when accompanied by compulsive motives, withdrawal‐like distress, loss of control, or continued exercise despite harm [14, 18]. Therefore, greater weekly exercise duration should not be treated as a standalone diagnostic sign. For clinical and public‐health practice, high weekly volume may prompt additional questions regarding motives, distress when exercise is reduced, training through injury, and interference with work, study, or relationships.

### 4.1 Limitations

Several limitations should be acknowledged. First, the cross‐sectional design precludes temporal or causal interpretation. Second, only 32 participants met the EDS‐21 dependence‐risk classification. The original 10‐covariate candidate set therefore corresponded to approximately 3.2 events per covariate, which limits the information available for multivariable estimation. Firth penalization was used to reduce small‐sample and sparse‐data bias, but it does not eliminate uncertainty. Estimates, particularly for marital status, remain imprecise. Third, the exploratory multinomial dependence‐risk contrast is subject to the same small‐cell limitation. Fourth, recruitment from exercise and sports facilities within one geographical context in Türkiye limits generalizability. Fifth, reliance on self‐report measures and the absence of eating disorder symptoms, body image disturbance, exercise motivation, social media/fitspiration exposure, and other potentially relevant personality characteristics limit clinical interpretation and prevent distinction between primary and secondary or instrumental exercise dependence. Sixth, depression symptom severity was low and showed restricted variability, raising the possibility of a floor effect. Finally, BMI and exercise duration were self‐reported and may contain measurement error. Future studies should use longitudinal designs, larger numbers of dependence‐risk cases, structured clinical interviews, objective or device‐based exercise measures where feasible, and validated measures of eating pathology, body image, exercise motives, social media exposure, and injury history.

## 5 Conclusion

In the present cross‐sectional study of regular exercisers, EDS‐21 dependence‐risk classification was associated with higher total impulsivity, single marital status, and longer weekly exercise duration in a Firth‐penalized multivariable model. Sensation seeking and positive urgency were comparatively stronger bivariate correlates, but they were not established as robust independent multivariable predictors. Depression symptom severity was not independently associated with the dependence‐risk classification. However, low scores and limited between‐group variability mean that this null finding should be interpreted cautiously. The marital‐status estimate was also imprecise and should be considered hypothesis‐generating. Because eating disorder symptoms, body image, and exercise motives were not assessed, the data cannot distinguish primary from secondary exercise dependence. Clinically, high exercise volume should not be pathologized in isolation. Assessment should focus on loss of control, withdrawal‐like distress, persistence despite harm, and functional impairment. Longitudinal and clinically enriched studies are needed to clarify temporal relationships and diagnostic significance.

## Author Contributions

**Beyza Nur Gürbüz:** conceptualization (lead), methodology (lead), formal analysis (lead), writing – original draft (lead), writing – review and editing (equal). **Hasan Durmuş:** methodology (supporting), supervision (lead), writing – review and editing (equal). **Yasin Kavla:** validation (lead), investigation (supporting), writing – review and editing (equal). **Mark D. Griffiths:** visualization, validation, writing – review and editing (equal). **Attila Szabo:** methodology, formal analysis, validation, writing – review and editing (equal).

## Funding

The authors have nothing to report.

## Ethics Statement

Ethical approval was obtained from the Erciyes University Ethics Committee (Approval No: 2025/196, Date: April 16, 2025). Signed written informed consent was obtained from all participants before survey completion.

## Conflicts of Interest

The authors declare no conflicts of interest.

## Declaration of AI Use

The authors used *Claude AI (Opus 4.6)* for grammar and language editing. All AI‐assisted text was reviewed and approved by the authors, who take full responsibility for the final content of the manuscript.

## Author Declaration

All authors have read and approved the final version of the manuscript. Beyza Nur Gürbüz had full access to all of the data in this study and takes complete responsibility for the integrity of the data and the accuracy of the data analysis.

## Transparency Statement

Beyza Nur Gürbüz, the manuscript guarantor, affirms that this manuscript is an honest, accurate, and transparent account of the study being reported; that no important aspects of the study have been omitted; and that any discrepancies from the study as planned (and, if relevant, registered) have been explained.

## Supporting information


Supporting File

## Data Availability

The data supporting the findings of this study are openly available in the Mendeley Data Repository at https://doi.org/10.17632/9zb9nrzrbc.1.
