# Supplementary material for: Exercise Dependence and Its Health Correlates: A Cross‐Sectional Survey‐Based Study of Impulsivity, Depression Symptom Severity, and Exercise Duration
Source: Health Sci Rep. 2026 Sep 7;9(9):e73162. doi: 10.1002/hsr2.73162 (PMC13548418; doi:10.1002/hsr2.73162)
Supplement: Supplementary file 1 [file HSR2-9-e73162-s001.docx]

**Exercise Dependence and Its Health Correlates: A Survey-Based Study of Impulsivity, Depression Symptom Severity, and Exercise Duration**

**Supplementary tables**

**Table S1. *Comparison of Exercise Dependence, Impulsivity, and Depression Scores by Gender***

| **Variables** | **Males**  **Median (Q1-Q3)** | **Females**  **Median (Q1-Q3)** | **Statistic** | ***p*** |
| --- | --- | --- | --- | --- |
| **EDS-21 (Total Score)** | 48.0 (37.0-60.0) | 42.0 (33.0-52.0) | *U* = 72418.5 | **< .001** |
| **UPPS Impulsivity (Total)** | 41.0 (36.0-47.0) | 41.5 (35.0-47.0) | *U* = 88065.5 | .534 |
| **BECK Depression** | 2.0 (1.0-4.0) | 3.0 (1.0-5.0) | *U* = 80421.5 | **.004** |
| **EDS-21 Classification** | **n (%)** | **n (%)** | *\chi^2* |  |
| *Asymptomatic* | 161 (37.1%) | 214 (51.4%) | 20.34 | **< .001** |
| *Symptomatic* | 252 (58.1%) | 191 (45.9%) |  |  |
| *Dependent* | 21 (4.8%) | 11 (2.6%) |  |  |

Note. U = Mann-Whitney U test statistic; χ² = Pearson chi-Square test statistic. Significant *p*-values (< .05) are bold.

**Table S2. *Prevalence of Primary Exercise Types Engaged in by the Participants***

| **Exercise Types** | **n** | **Percentage of Total Sample (N = 850)** |
| --- | --- | --- |
| Fitness/Gym | 589 | 69.3% |
| Swimming | 479 | 56.4% |
| Walking | 475 | 55.9% |
| Running | 245 | 28.8% |
| Cycling | 169 | 19.9% |
| Pilates/Yoga | 98 | 11.5% |
| Combat sports/Martial arts | 50 | 5.9% |

*Note.* Participants could select more than one exercise type; therefore, the sum of percentages exceeds 100%.
